# Supplementary material for: Sex-based disparities in DNA methylation and gene expression in late-gestation mouse placentas
Source: Biol Sex Differ. 2024 Jan 6;15:2. doi: 10.1186/s13293-023-00577-w (PMC10770955; doi:10.1186/s13293-023-00577-w)
Supplement: Supplementary file 3 — Additional file 3: Figure S1. Expression of Y chromosome genes in male placentas A) Differential expression analysis of Y chromosome genes in male and female E18.5 placentas (n = 121). Colored dots represent statistically significant DEGs (p < 0.05; n = 7) B) Expression levels (z-scores) of the Y chromosome DEGs. Figure S2. DEG abundance in male and female placentas A–B) MA plot of the differential expression (log2 fold change) values of normalized read counts and their average expression levels in A) male and B) female E18.5 placentas. The plots include all analyzed genes (left; n = 29,480); genes with read counts > 10,000 (middle; n = 29,165 and 29,160 in male and female placentas, respectively), and genes with read counts > 100 (right; n = 18,160 and 18,173 in male and female placentas, respectively). Colored dots represent significant DEGs on autosomes (pink) and X chromosomes (blue). The dotted black rectangle indicates the subset of the graph shown on the right. C) Differential expression values of each analyzed gene) in male and female placentas relative to their promoter’s GC content (%). Significant DEGs on autosomes and X chromosomes are shown in pink and blue, respectively. Figure S3. DMRs occur throughout the E18.5 placenta genome but are concentrated on the X chromosomes A) DNA methylation levels of a random subset of tiles in the individual male and female placenta samples. B) Mean DNA methylation levels within ± 15 kb of a transcriptional start site (TSS) or transcriptional end site (TES) in all analyzed tiles from male and female placentas. C) Distributions of the DNA methylation levels of various chromosomes in male and female placentas. Median DNA methylation values are indicated with diamonds. D) DNA methylation levels in tiles associated with autosomes (top) and X chromosomes (bottom) in male and female placentas. ****p < 0.0001, ***p < 0.001 by two-proportion z-test. E) Average DNA methylation levels in tiles associated with various genomic feat [file 13293_2023_577_MOESM3_ESM.docx]

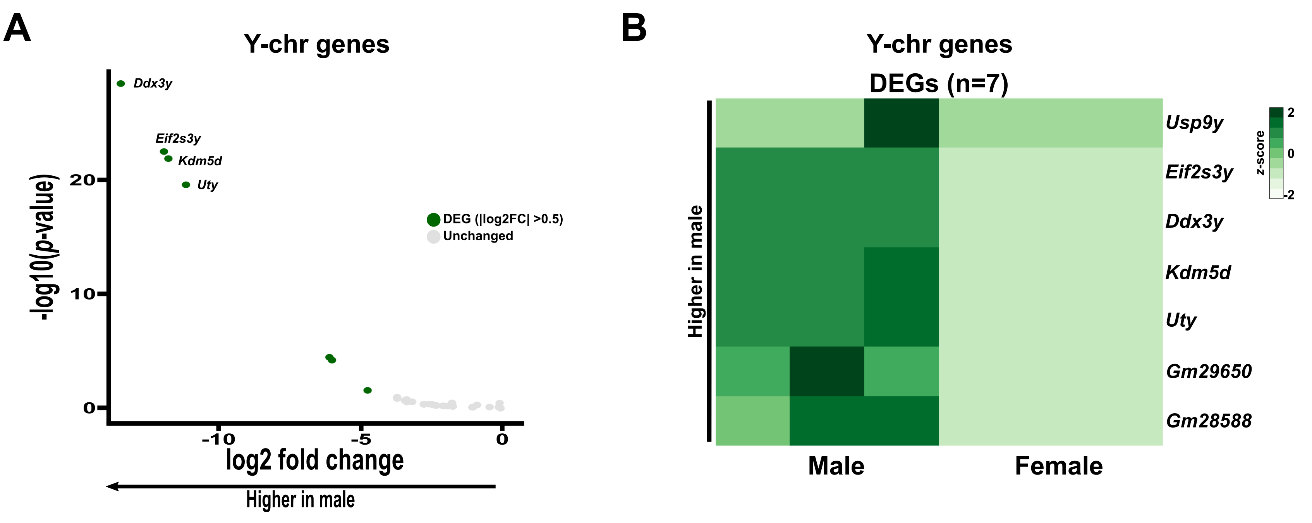


**Figure S1. Expression of Y chromosome genes in male placentas**

**
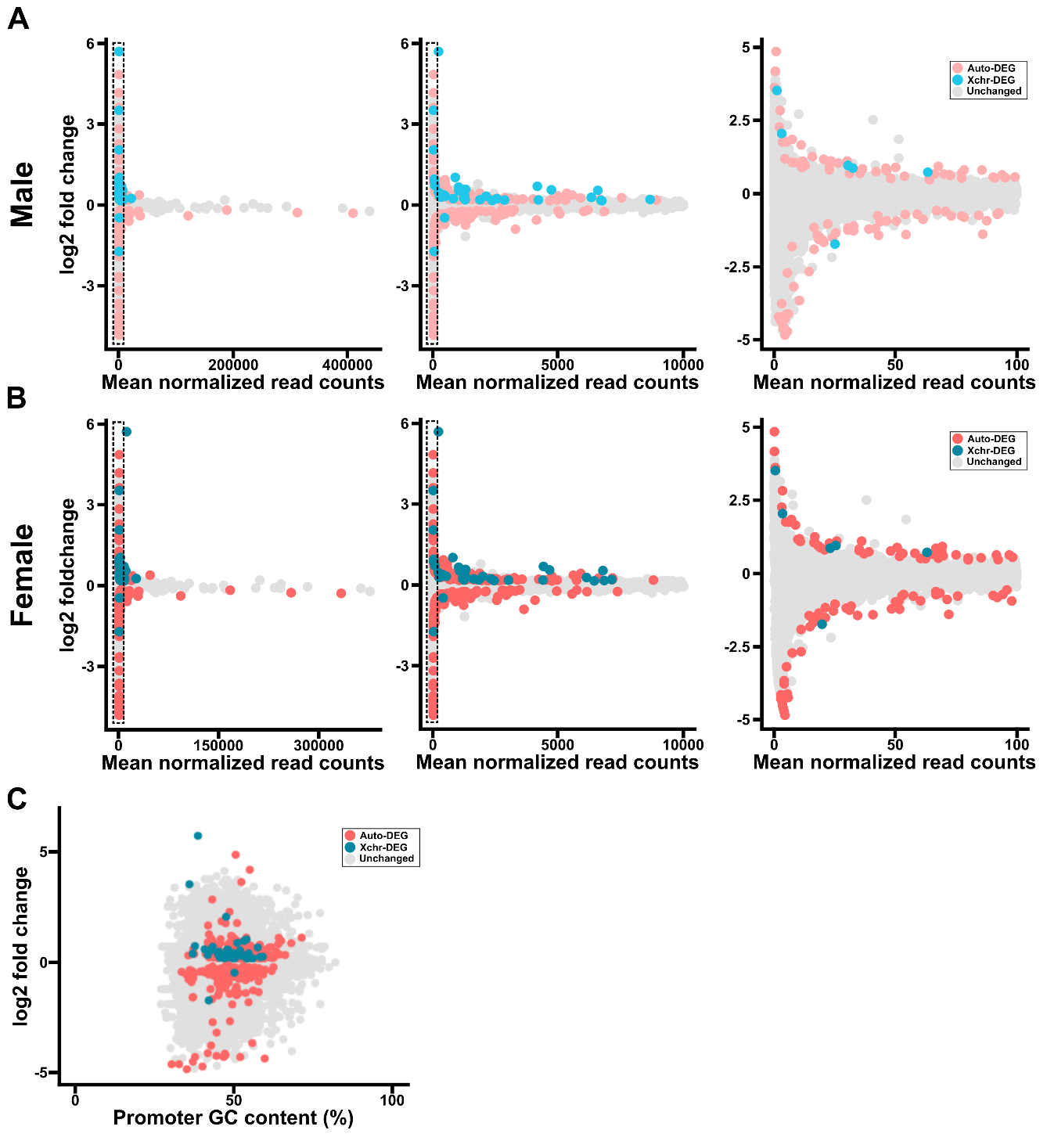
**

**Figure S2.** **DEG abundance in male and female placentas**

**
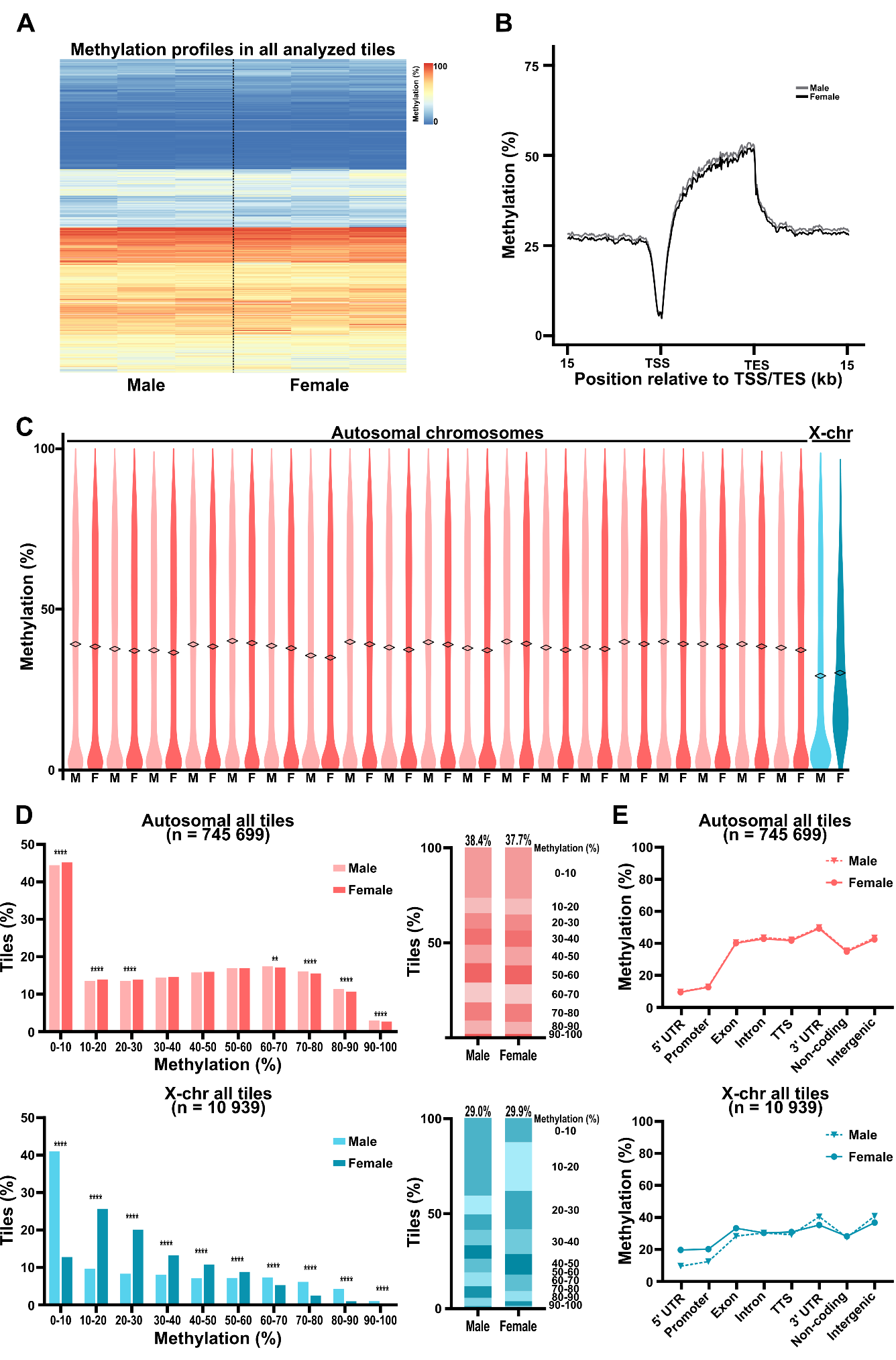
**

**Figure S3. DMRs occur throughout the E18.5 placenta genome but are concentrated on the X chromosomes**

**
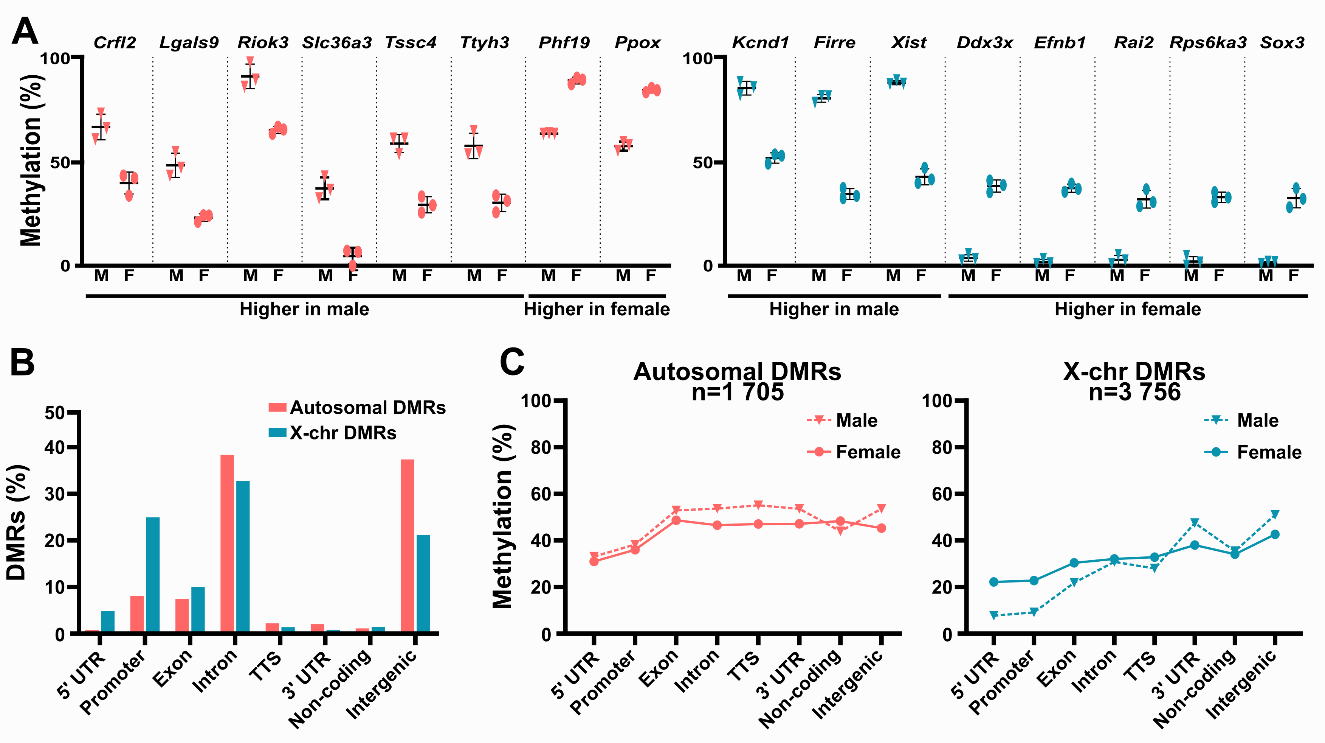
**

**Figure S4. DNA methylation differences in various genomic elements**

**
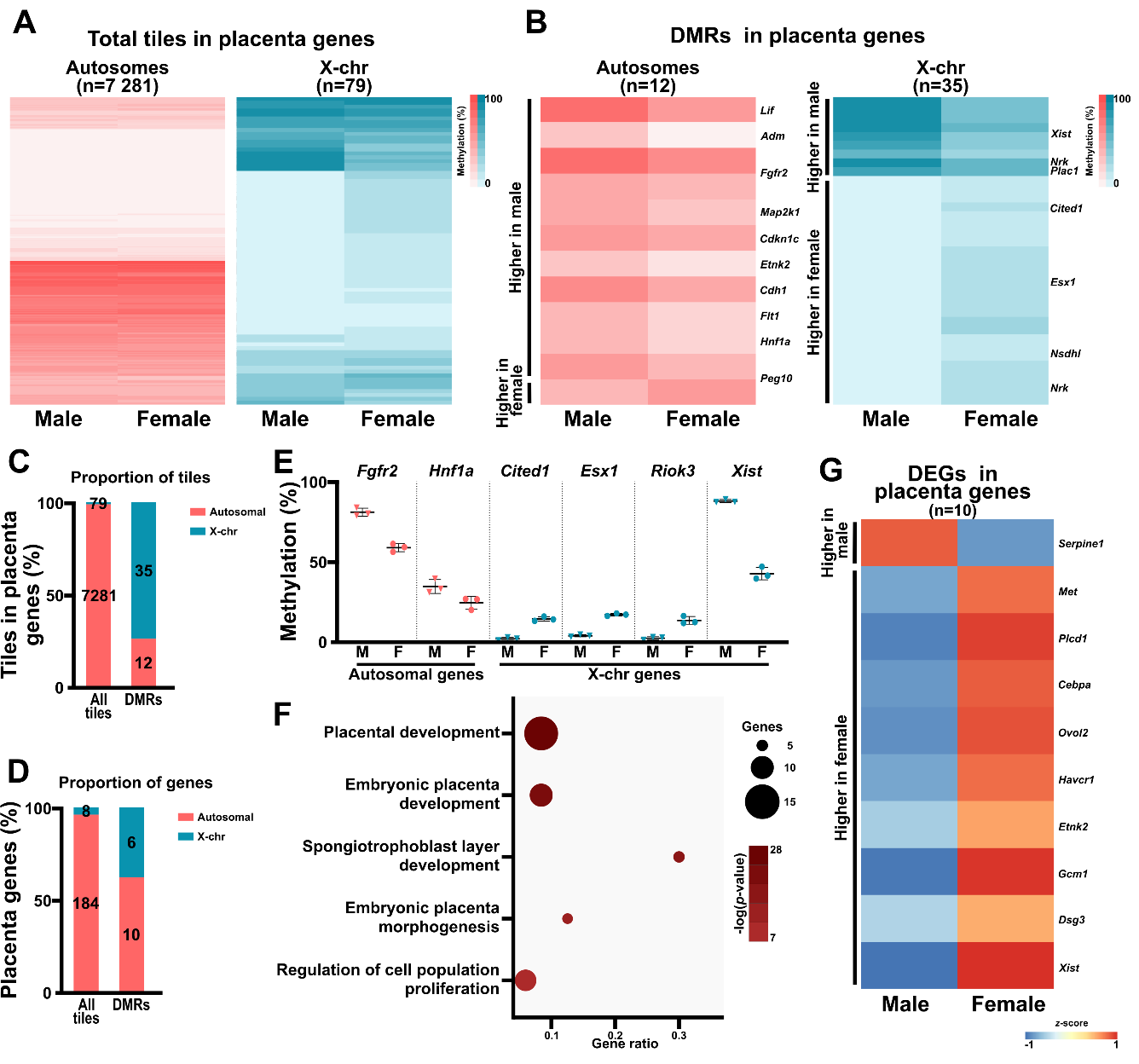
**

**Figure S5. DNA methylation and expression patterns of genes essential for placental development**
